# Postnatal Changes in Humerus Cortical Bone Thickness Reflect the Development of Metabolic Bone Disease in Preterm Infants

**DOI:** 10.1155/2016/2176594

**Published:** 2016-04-18

**Authors:** Shuko Tokuriki, Aiko Igarashi, Takashi Okuno, Genrei Ohta, Takuya Kosaka, Yusei Ohshima

**Affiliations:** Department of Pediatrics, Faculty of Medical Sciences, University of Fukui, 23-3 Shimoaizuki, Matsuoka, Eiheiji-cho, Yoshida-gun, Fukui 9101193, Japan

## Abstract

*Objective*. To use cortical bone thickness (CBT) of the humerus to identify risk factors for the development of metabolic bone disease in preterm infants.* Methods*. Twenty-seven infants born at <32 weeks of gestational age, with a birth weight of <1,500 g, were enrolled. Humeral CBT was measured from chest radiographs at birth and at 27-28, 31-32, and 36–44 weeks of postmenstrual age (PMA). The risk factors for the development of osteomalacia were statistically analyzed.* Results*. The humeral CBT at 36–44 weeks of PMA was positively correlated with gestational age and birth weight and negatively correlated with the duration of mechanical ventilation. CBT increased with PMA, except in six very early preterm infants in whom it decreased. Based on logistic regression analysis, gestational age and duration of mechanical ventilation were identified as risk factors for cortical bone thinning.* Conclusions*. Humeral CBT may serve as a radiologic marker of metabolic bone disease at 36–44 weeks of PMA in preterm infants. Cortical bones of extremely preterm infants are fragile, even when age is corrected for term, and require extreme care to lower the risk of fractures.

## 1. Introduction

Metabolic bone disease (MBD) has been identified as one of the health problems associated with prematurity and results from mineral deficiency [1–3]. Radiological changes, including characteristics of rickets, are identifiable in 55% of infants weighing <1000 g at birth and in 23% of those weighing <1500 g [1]. These infants are at an increased risk for fractures. The optimal mineral and vitamin D supplementation to prevent MBD in premature infants is fortified breast milk or formula, high calcium (Ca), phosphate (P), and vitamin D contents [2]. Despite supplementation, there is accumulating evidence of reduced bone mineral density (BMD) in preterm infants, even when age is corrected for term [3–5].

BMD measurements using dual-energy X-ray absorptiometry (DXA) are generally used to diagnose MBD and to assess the risk for fracture. The determination of measurements obtained by DXA tends to be variable, however, as they are influenced by the type of device, the measurement mode, and the analysis software. Moreover, preterm infants cannot easily undergo DXA measurement without sedation. Therefore, DXA may not be useful for the measurement of BMD of premature infants, especially during the early postnatal period when respiration and circulation are unstable.

The radiographic measurements of cortical bone thickness (CBT) have been used to evaluate osteoporosis in adults [6, 7], with CBT measures significantly correlating with the BMD of the femur and lumbar vertebrae measured by DXA. Although infants with MBD show radiographic evidence of metaphyseal splaying, cupping, and fraying of tubular bones [8], the association between pathophysiological findings of humeral CBT in preterm infants and the development of MBD remains unclear. Therefore, the aim of our study was to examine the relationship between humeral CBT and presumed precipitating factors for MBD, including gestational age, birth weight, duration of mechanical ventilation, administered medication, nutrition, and complications. We also analyzed the postnatal changes in CBT of preterm infants to identify the risk factors for decreasing BMD and the development of MBD.

## 2. Patients and Methods

### 2.1. Patients Selection

Preterm infants born at <32 weeks of gestational age and with a birth weight of <1,500 g, who were admitted to the Neonatal Intensive Care Unit (NICU) at the University of Fukui Hospital, between December 2008 and June 2015, were enrolled in this study. Infants with congenital malformations, chromosome abnormalities, and metabolic disease were excluded. The study was approved by the Institutional Ethics Committee at the University of Fukui. Parents provided written consent for their infants to participate in the study.

### 2.2. Parenteral Nutrition Intervention and Enteral Nutrition

In our NICU, parenteral nutrition (PN) was initiated in preterm infants within the first day of life. Calcium (Ca, 37–75 mg/kg/day) was routinely added to PN solutions, with phosphate (P, 15–30 mg/kg/day) subsequently added within a week. PN solutions were also supplemented with 40–80 IU/kg/day of vitamin D_2_. Enteral feeding was initiated as soon as possible within the first postnatal week. Mother's milk was preferred; when each mother's milk volume reached 100 mL/kg/day, breast milk was fortified with 1.3 g/100 mL of HMS-1 (Morinaga Milk Industry Co., Ltd., Tokyo, Japan), which contains 78 kcal, 2.0 g of protein, 99 mg of Ca, and 57 mg of P/100 mL, as well as 10–30 mg/kg/day of P supplements. When mother's milk was insufficient or unavailable, Meiji LW preterm formula was used (Meiji Dairies Co., Tokyo, Japan), which contains 70 kcal, 2.0 g of protein, 65 mg of Ca, and 41 mg of P/100 mL. During the study period, we adjusted the daily intake of Ca, P, and vitamin D_2_ to maintain the serum Ca levels between 8.2 and 9.8 mg/dL, serum P levels between 5.0 and 7.0 mg/dL, urinary Ca/creatinine (Cr) between 0.3 and 0.5 mg/mg Cr, and urinary P/Cr between 0.5 and 1.0 mg/mg Cr.

### 2.3. Cortical Bone Thickness Measurements

CBT measures were calculated from chest radiographs routinely obtained to assess lung condition at birth at 27-28, 31-32, and 36–44 weeks of PMA and a body weight >2,000 g. Chest radiographs were retrospectively retrieved from the radiology database of the University of Fukui Hospital. Radiographs were obtained using a Siemens Mobilett XP Eco system (Siemens Healthcare GmbH, Henkestr, Erlangen, Germany), with a tube voltage of 60 kV and current time product of 1.6 mAs.

**Figure 1:**
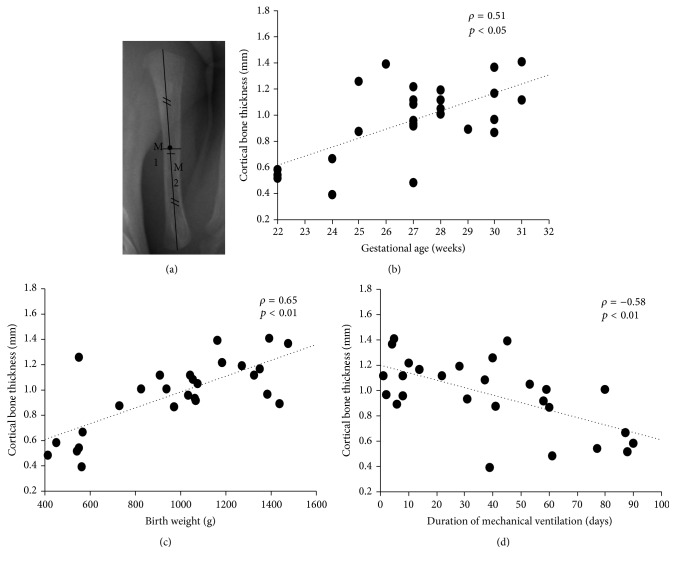
The humeral cortical bone thickness was measured at the middle of the diaphysis (a).* M*1, the breadth of the bone shaft;* M*2, the width of the intramedullary canal. Correlation between humeral cortical bone thickness and gestational age (b), birth weight (c), and the duration of mechanical ventilation (d).

The medial and lateral CBTs of the humeral diaphysis were calculated by adapting the methods previously reported by Tingart et al. [6] and Mather et al. [7] for our needs. CBT of the humeral diaphysis was measured using the digital measurement tool on the picture archiving and communication system, which has a precision of ±0.01 mm (Figure 1(a)). For measurement, a perpendicular line was drawn from the medial outer cortex to the lateral outer cortex at the middle of the humeral diaphysis and the interline distance measured by digital caliper to provide the breadth of the bone shaft (*M*1). The width of the intramedullary canal on the same line was determined (*M*2). CBT was calculated by subtracting* M*2 from* M*1 and dividing by 2. All measurements were made by observers who were blinded to the infants' background.

### 2.4. Data Collection

Demographic and clinical data regarding the infants' perinatal and postnatal courses were extracted from the medical records. Serum Ca, P, and alkaline phosphatase (ALP) levels, and urinary Ca/Cr, P/Cr and tubular reabsorption of P levels were determined at a PMA of 36 to 44 weeks.

The independent variables were gestational age; birth weight; small for gestational age (SGA) status; sex; prenatal steroid use; duration of mechanical ventilation; duration of intravenous sedation; postnatal complications, such as respiratory distress syndrome, patent ductus arteriosus (PDA), intraventricular hemorrhage, cystic periventricular leukomalacia, necrotizing enterocolitis, early and late hemoculture positive for sepsis, bronchopulmonary dysplasia (BPD), retinopathy of prematurity, and fracture; treatments administrated, such as total dosage of hydrocortisone, diuretic drug use, number of days with enteral feeding >100 mL/kg/day, and Ca/P/vitamin D supply at that point; PMA; and body weight at the time of radiographic evaluations. PDA was defined as a requirement for indomethacin treatment and/or surgical closure. BPD was defined as oxygen dependence at 28 days of life.

### 2.5. Statistical Analysis

The Mann-Whitney *U* and Fisher exact probability tests were used to analyze the data. Correlations were evaluated using the nonparametric Spearman rho test. The data are presented as medians (ranges). A *p* value < 0.05 was denoted to be a statistically significant difference. Stepwise logistic regression analysis was performed using SPSS software, version 14.0 (IBM, Armonk, NY, USA). To establish a final regression model, a stepwise downward selection model was used, starting with a prespecified set of candidate variables. An entry criterion of *p* < 0.05 was used for statistical significance during this process.

## 3. Results

During the study period, 555 infants were admitted to our NICU and 70 infants had a gestational age of less than 32 weeks and a birth weight of less than 1,500 g. Twelve infants were excluded from our study because of death, chromosomal abnormality, or hospital transfer before 36 weeks of PMA. In 27 out of the remaining 58 infants, eligible chest radiographs, including the whole length of right or left humerus, had been obtained at 36–44 weeks of PMA and at the time point of body weight >2,000 g. Among this group, 25 infants also had adequate chest radiographs obtained at birth. In addition, some infants had undergone radiographic evaluation at two different times, namely, at 27-28 and 31-32 weeks of PMA. The demographic data of the 27 infants included in our analysis are listed in Table 1.

Humeral CBT at 36–44 weeks of PMA was positively correlated to gestational age (*ρ* = 0.51, *p* < 0.05) and birth weight (*ρ* = 0.65, *p* < 0.01) and negatively correlated to duration of mechanical ventilation (*ρ* = −0.58, *p* < 0.01; Figures 1(b), 1(c), and 1(d)).

**Figure 2:**
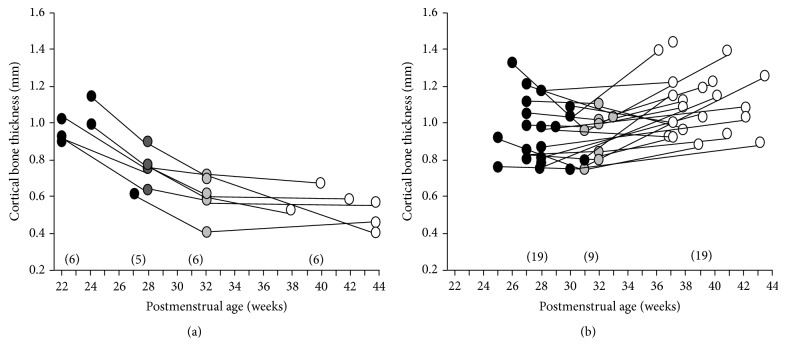
Chronological changes in humeral cortical bone thickness in the infants with (a) or without (b) a 20% or more decrease in cortical bone thickness between birth and 36–44 weeks of PMA (black circles, at birth; dark circles, 27-28 weeks of PMA; gray circles, 31-32 weeks of PMA; white circles, 36–44 weeks of PMA). The number of subjects of radiographic evaluation is shown in parentheses.

For the majority of infants, CBT increased as with PMA. In six infants, however, the CBT gradually decreased by 20% or more, from birth to 36–44 weeks of PMA (Figure 2). Comparative measures for these groups of infants are reported in Table 2. Compared with the former 19 infants, the latter six infants with decreasing CBT had a younger gestational age (median [range] 28 weeks [25–31 weeks] versus 23 weeks [22–27 weeks], *p* < 0.01), lower birth weight (1064 g [552–1474 g] versus 545 g [412–568 g], *p* < 0.01), and longer mechanical ventilation period (31 days [1–80 days] versus 82 days [39–90 days], *p* < 0.01). Bone fractures occurred in two infants in the decreasing CBT group. These infants were born at a gestational age of 22 weeks and their fractures were recognized at 87 and 103 days of age.

Serum ALP levels and urinary P/Cr levels of infants in the decreasing CBT group were significantly higher than those in the nondecreasing CBT group. There were no significant between-group differences in serum Ca and P levels or urinary Ca/Cr levels at 36–44 weeks of PMA (Table 3).

The risk factors for decreasing CBT were evaluated by univariate logistic regression analysis. Gestational age (*p* = 0.017, odds ratio (OR), 0.27) and duration of mechanical ventilation (*p* = 0.017, OR, 1.09) were identified as significant risk factors, but not birth weight or total dosage of hydrocortisone (Table 4). Finally, multivariate logistic regression analysis identified gestational age as a sole risk factor for decreasing CBT.

## 4. Discussion

In the present study, we demonstrated that the risk factors of MBD, a younger gestational age, lower birth weight, and longer duration of mechanical ventilation, are also associated with decreasing humeral CBT. As CBT values at birth were comparable across all infants in our study group, our findings underline the importance of continued monitoring of CBTs in preterm infants, regardless of gestational age and birth weight.

MBD is diagnosed by radiological changes and its severity classified by the radiological characteristics observed in rickets [8]. However, there is no gold standard for the diagnosis of the early stage of MBD. BMD assessed by DXA has been reported to reflect the state of bone mineralization. Lumbar vertebrae are generally used to measure BMD by DXA. Since 80% of human bone mass consists of cancellous bones, lumbar vertebrae are also suitable for estimating the total bone mass. Since premature infants routinely undergo DXA assessment at discharge, there is lack of data on BMD of premature infants during the early postnatal period.

The incidence of bone fractures in preterm infants has been reported to be between 1.2% and 10.5% [9–11]. Most bone fractures in preterm infants, without a traumatic cause, occur in the ribs and long bones of the extremities and are detected between 33 and 129 days of postnatal age [11]. In our study, the two infants with fractures of the long bones of the extremities exhibited a large decrease in humeral CBT. Although not all very early preterm infants suffer from bone fracture, the decrease in CBT may be useful to predict the risk of fracture. Therefore, assessment of the CBT of the humeral diaphysis by radiographs is a very simple technique, which can be used to screen for preterm infants at higher risk for fracture. If routine chest radiographs include imaging of at least one humerus over its entire length, then CBT measures can be readily obtained.

Fetal bone mineralization occurs during the last trimester of gestation, with deposition of 80% of the mineral contents of term newborns [12]. Therefore, preterm infants born prior to the last trimester require sufficient Ca and P supplementation to be provided through PN. Rigo et al. recommended an intake of 100 to 160 mg/kg/day of Ca and 60 to 90 mg/kg/day of P in order to decrease the risk for osteopenia and fracture [2]. Pereira-Da-Silva et al. reported that early parenteral administration of 75 mg/kg/day of Ca and 44 mg/kg/day of P prevented decrease in bone strength of preterm infants [13]. The administered doses of Ca and P were slightly lower than the above recommended doses. Serum Ca and P levels at 36–44 weeks of PMA were almost within the normal range, whereas urinary P/Cr levels of the decreasing CBT group tended to be higher than those of the nondecreasing CBT group. Although there was no apparent difference in the administered doses of Ca, P, and vitamin D between the two groups, the doses might be insufficient for some very early preterm infants, especially the younger and smaller ones.

Catache and Leone demonstrated that increased urinary Ca concentration reflected the development of MBD [14]. Urinary Ca and P or the Ca/P ratio has been shown to correlate with bone mineralization in preterm infants [15, 16]. However, urinary excretion of Ca and P is influenced by several factors, including feeding and medications. The adjustment of administered Ca and P based on serum Ca and P and urinary Ca/Cr and P/Cr resulted in lower dosages than the recommended ones. Although radiological changes of CBT do not appear in early stage of MBD, continued monitoring of CBT could be a reliable marker in concert with urinary and serum biochemical markers.

A possible limitation of this study is the small sample size. Since chest radiographs were retrospectively analyzed, one-third of the preterm infants failed to undergo chest radiography that included the whole length of the humerus. Additionally, we could not assess the correlation between the BMD measured by DXA and cortical bone thickness.

## 5. Conclusions

In conclusion, our data indicate that humeral CBT gradually increases after birth and that it may be a reliable predictor of ongoing MBD in very premature infants. Attention is needed in handling very early premature infants with decreasing CBT to reduce the risk for fractures. It is also imperative that doses of Ca, P, and vitamin D supplementation are adjusted for each infant to optimize bone development over the early postnatal period and likely beyond.

## Figures and Tables

**Table 1:** Demographic and clinical characteristics of the study subjects (*n* = 27).

Characteristic	Value
Male sex, *n* (%)	15 (55.6)
Gestational age (weeks)	27 (22–31)
Birth weight (g)	1041 (412–1474)
SGA (<tenth percentile), *n* (%)	5 (18.5)
Days of enteral feeding > 100 mL/kg/day (day)	17 (7–55)
Calcium administration (mg/kg/day)	45.9 (20.7–66.0)
Phosphorus administration (mg/kg/day)	33.1 (14.5–61.3)
Vitamin D administration (IU/kg/day)	137.5 (13.3–212.0)
Prenatal steroid use, *n* (%)	25 (92.6)
Total dosage of hydrocortisone (mg/kg)	21.4 (0.0–56.8)
Diuretic drug use, *n* (%)	13 (48.1)
Duration of mechanical ventilation (days)	39 (1–90)
Duration of sedation (days)	10 (0–72)
RDS, *n* (%)	24 (88.9)
PDA, *n* (%)	9 (33.3)
IVH grade III or IV, *n* (%)	3 (11.1)
cPVL, *n* (%)	2 (7.4)
NEC, *n* (%)	1 (3.7)
Sepsis (hemoculture positive), *n* (%)	1 (3.7)
BPD (oxygen at 28 days of life), *n* (%)	20 (74)
ROP stage ≥ 2, *n* (%)	13 (48.1)
Fracture, *n* (%)	2 (7.4)

BPD, bronchopulmonary dysplasia; cPVL, cystic periventricular leukomalacia; IVH, intraventricular hemorrhage; NEC, necrotizing enterocolitis; PDA, patent ductus arteriosus; PMA, postmenstrual age; RDS, respiratory distress syndrome; ROP, retinopathy of prematurity; SGA, small for gestational age.

Values are expressed as median (range) or number (%).

**Table 2:** Comparison of demographic data of preterm infants with and without decreasing cortical bone thickness at 36–44 weeks of postmenstrual age.

Characteristic	Decreasing cortical bone thickness (*n* = 6)	Nondecreasing cortical bone thickness (*n* = 19)	*p* value
Cortical bone thickness at birth (mm)	0.96 (0.62–1.15)	0.96 (0.73–1.33)	0.95
Cortical bone thickness at 36–44 weeks of PMA	0.53 (0.40–0.67)	1.05 (0.87–1.39)	**<0.01**
Male sex, *n* (%)	3 (50.0)	11 (57.9)	>0.99
Gestational age (weeks)	23 (22–27)	28 (25–31)	**<0.01**
Birth weight (g)	545 (412–568)	1064 (552–1474)	**<0.01**
SGA (<tenth percentile), *n* (%)	1 (16.7)	4 (21.0)	>0.99
Days of enteral feeding > 100 mL/kg/day (day)	24 (11–32)	16 (7–55)	0.19
Calcium administration (mg/kg/day)	28.0 (20.7–50.0)	45.9 (20.7–57.5)	0.48
Phosphorus administration (mg/kg/day)	29.1 (14.5–39.6)	33.1 (15.5–61.3)	0.34
Vitamin D administration (IU/kg/day)	166.5 (29.2–212.0)	137.5 (13.3–211.2)	0.80
Total dosage of hydrocortisone (mg/kg)	28.7 (12.0–45.4)	20.5 (0.0–56.8)	0.14
Diuretic drug use, *n* (%)	5 (83.3)	8 (42.1)	0.16
Duration of mechanical ventilation (days)	82 (39–90)	31 (1–80)	**<0.01**
Duration of intravenous sedation (days)	16 (1–72)	10 (0–55)	0.80
PDA, *n* (%)	3 (50.0)	6 (31.9)	0.63
NEC, *n* (%)	1 (16.7)	0 (0)	0.24
Sepsis (hemoculture positive), *n* (%)	1 (16.7)	0 (0)	0.24
BPD (oxygen at 28 days of life), *n* (%)	6 (100)	14 (73.7)	0.30
Fracture, *n* (%)	2 (33.3%)	0 (0)	**0.05**

BPD, bronchopulmonary dysplasia; cPVL, cystic periventricular leukomalacia; IVH, intraventricular hemorrhage; NEC, necrotizing enterocolitis; PDA, patent ductus arteriosus; PMA, postmenstrual age; RDS, respiratory distress syndrome; ROP, retinopathy of prematurity; SGA, small for gestational age.

Values are expressed as median (range) or number (%).

**Table 3:** Comparison of the serum and urinary biochemical data of preterm infants with and without decreasing cortical bone thickness at 36–44 weeks of postmenstrual age.

Characteristic	Decreasing cortical bone thickness (*n* = 6)	Nondecreasing cortical bone thickness (*n* = 19)	*p* value
Serum Ca level (mg/dL)	9.7 (9.3–10.1)	9.6 (9.0–10.7)	0.80
Serum P level (mg/dL)	5.9 (4.7–6.0)	5.8 (4.8–6.6)	0.97
Urinary Ca/Cr level (mg/mg)	0.3 (0.1–0.6)	0.4 (0.1–1.3)	0.46
Urinary P/Cr level (mg/mg)	1.6 (0.2–2.5)	0.5 (0.0–1.3)	**0.03**
TRP (%)	94.4 (85.0–99.0)	98 (90–100)	0.07
ALP (U/L)	1676 (1265–2692)	1036 (679–1877)	**<0.01**

ALP, alkaline phosphatase; Ca, calcium; Cr, creatinine; P, phosphorus; PMA, postmenstrual age; TRP, tubular reabsorption of phosphate. Values are expressed as median (range) or number (%).

**Table 4:** Risk factors of decreasing humoral cortical bone thickness in preterm infants at 36–44 weeks of postmenstrual age.

Predictors	Univariate analysis			Multivariate analysis		
	Unadjusted OR	95% CI	*p* value	Adjusted OR	95% CI	*p* value
The initial model						
Gestational age	0.27	0.09–0.80	**0.017**	0.00	0.000–1.54*E* + 013	0.560
Birth weight	0.97	0.91–1.04	0.366	0.99	0.87–1.13	0.913
Mechanical ventilation	1.09	1.02–1.17	**0.017**	2.08	0.00–15971.43	0.873
Total dosage of hydrocortisone	1.04	0.98–1.11	0.188	5.25	0.000–142837759	0.849

The final model						
Gestational age				**0.27**	**0.09**–**0.80**	**0.017**

PMA, postmenstrual age; OR, odds ratio; CI, confidence interval.
